# Heparinized silk fibroin hydrogels loading FGF1 promote the wound healing in rats with full-thickness skin excision

**DOI:** 10.1186/s12938-019-0716-4

**Published:** 2019-10-02

**Authors:** Sirong He, Dan Shi, Zhigang Han, Zhaoming Dong, Yajun Xie, Fengmei Zhang, WenXin Zeng, Qiying Yi

**Affiliations:** 1 Department of Immunology, College of Basic Medicine, Chongqing Medical University, Chongqing, 400016 China; 2 Intervention Section, Chinese Medicine Hospital of Dianjiang County, Chongqing, 408300 China; 3 Laboratory Animal Center, Chongqing Medical University, Chongqing, 400016 China; 4 Biological Science Research Center, Southwest University, Chongqing, 400716 China; 5 Chongqing Engineering and Technology Research Center for Novel Silk Materials, Southwest University, Chongqing, 400716 China; 6 The M.O.E. Key Laboratory of Laboratory Medical Diagnostics, The College of Laboratory Medicine, Chongqing Medical University, Chongqing, 400016 People’s Republic of China

**Keywords:** Silk fibroin, Hydrogel, FGF1, Wound healing

## Abstract

**Background:**

Silk fibroin hydrogel, derived from *Bombyx mori* cocoons, has been shown to have potential effects on wound healing due to its excellent biocompatibility and less immunogenic and biodegradable properties. Many studies suggest silk fibroin as a promising material of wound dressing and it can support the adhesion and proliferation of a variety of human cells in vitro. However, lack of translational evidence has hampered its clinical applications for skin repair. Herein, a heparin-immobilized fibroin hydrogel was fabricated to deliver FGF1 (human acidic fibroblast growth factor 1) on top of wound in rats with full-thickness skin excision by performing comprehensive preclinical studies to fully evaluate its safety and effectiveness. The wound-healing efficiency of developed fibroin hydrogels was evaluated in full-thickness wound model of rats, compared with the chitosan used clinically.

**Results:**

The water absorption, swelling ratio, accumulative FGF1 releasing rate and biodegradation ratio of fabricated hydrogels were measured. The regenerated fibroin hydrogels with good water uptake properties rapidly swelled to a 17.3-fold maximum swelling behavior over 12 h and a total amount of 40.48 ± 1.28% hydrogels was lost within 15 days. Furthermore, accumulative releasing data suggested that heparinized hydrogels possessed effective release behavior of FGF1. Then full-thickness skin excision was created in rats and left untreated or covered with heparinized fibroin hydrogels-immobilized recombinant human FGF1. The histological evaluation using hematoxylin and eosin (HE) and Masson’s trichrome (MT) staining was performed to observe the dermic formation and collagen deposition on the wound-healing site. To evaluate the wound-healing mechanisms induced by fibroin hydrogel treatment, wound-healing scratch and cell proliferation assay were performed. it was found that both fibroin hydrogels and FGF1 can facilitate the migration of fibroblast L929 cells proliferation and migration.

**Conclusion:**

This study provides systematic preclinical evidence that the silk fibroin promotes wound healing as a wound-healing dressing, thereby establishing a foundation toward its further application for new treatment options of wound repair and regeneration.

## Background

The normal wound-healing process is divided into several predictable phases: hemostasis, inflammation, proliferation and new tissue remodeling [1]. Silk fibroin from the cocoons of silkworm, composed of heavy and light chains linked by a disulfide bond, has been widely explored as wound dressing biomaterials for skin repair due to its hemostatic properties, low inflammatory potential, gaseous permeation, waterproofness and biodegradability of the material to escape disrupting the wound site [2–5]. It has been shown that silk fibroin can provide good structural support for cell attachment spreading, proliferation and migration of keratinocytes and fibroblasts [6, 7]. Several studies have reported that silk fibroin in different forms such as sponges, films and hydrocolloid dressings has been explored for its therapeutic effect in wound dressing to promote the healing process [8–10]. Some of them were widely used in growth factors, controlled release carrier of drugs and scaffolds of cell culture [11, 12]. It was implicated that regenerated silk fibroin hydrogel, a state of sol–gel transition, induced the healing process of different wound healing by increasing the cells proliferation and migration of various cells type. Moreover, the incorporation of growth factors into silk fibroin hydrogel can accelerate the wound-healing process. Despite the positive results for various silk biomaterials in vivo and in vitro studies to look for adequate evidence that are implicated in the wound-healing effects in different types of cells during the deposition of collagen and wound construction, however, little work has been done using silk fibroin hydrogel to support its potential application as a wound dressing in the biomedical field [13–17].

In the present study, silk fibroin hydrogel was firstly prepared and the release rate of the FGF1 from fibroin hydrogels was evaluated by immunodetection over a period of 5 days. The fibroin hydrogel properties such as water absorption, swelling behavior and in vitro degradation rate were analyzed in neutral conditions (in PBS, pH 7.4) at different time points. Moreover, the external morphology and porosity of silk fibroin hydrogel was observed by scanning electron microscopy. Secondly, full-thickness excisional wounds in SD (Sprague–Dawley) rats treated with silk fibroin hydrogels with or without FGF1 were used to evaluate in vivo wound-healing efficacy in comparison to commercial wound dressing product (positive control). The HE staining results demonstrated that fibroin hydrogels with FGF1 accelerated the dermis formation and epidermal differentiation into hair follicles and sebaceous glands at days 7 and 14. Furthermore, collagen deposition within fibroin hydrogels loading FGF1 was more stout and wavy, attributed to small scar healing. It has been demonstrated that FGF1 contributes to multiple cellular targets in vitro or diverse therapeutically relevant biological activities in animal models of tissue repair such as wound healing [18, 19]. Thirdly, proliferation and migration of the L929 cells seeded on the surface of fibroin hydrogel directly and cultured for 24 h in the presence or absence of FGF1 were measured by EdU (5-ethynyl-20-deoxyuridine) labeling and “scratch” assay wound model. These results suggested that fibroin hydrogel has tremendous potential for wound healing in the field of tissue regeneration engineering.

## Result

### Regenerated silk fibroin hydrogel preparation and properties characteristics

**Fig. 1:**
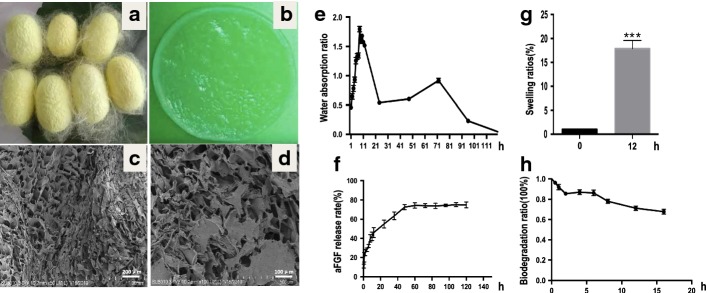
Fabrication and characteristic of the silk fibroin hydrogels. **a** Photos of silkworm cocoons from P50 strain. **b** Macroscopic appearance of silk fibroin hydrogel. **c**, **d** SEM images of pure silk fibroin hydrogel cross section. **e** Ratios of the time-dependent water absorption of fibroin hydrogel in PBS (pH 7.4) at room temperature. **f** In vitro release profile of FGF1 in fibroin hydrogels over 5 days at 37 °C. **g** Swelling ratio of FGF1for fibroin hydrogels in PBS (pH 7.4) at 37 °C. **h** Weight loss of fibroin at different degradation time points in PBS (pH 7.4), respectively. Data are shown as mean ± SD (*n* = 6)

The fabricated fibroin hydrogels from the cocoons of silkworm were thin and transparent (Fig. 1a, b). The porosity and pore size of fibroin hydrogels measured using surface area and pore characterization system (ASAP 3020; Micromeritics Instrument Corporation Tristar) were 92.0627% and 8.9605 μm, respectively. Scanning electron microscope (SEM) was used to study the morphology of the silk fibroin hydrogels with lamellar, porous and interconnected microstructures (Fig. 1c, d), which may allow the cells to move within the scaffold in the process of wound healing and regeneration. The water absorption ratio of fibroin hydrogels was measured at different time points after soaking in PBS (pH 7.4) solution. The ratio of water absorption was gradually increased to its peak by 8 h and decreased rapidly over the remaining 10 h (Fig. 1e). The fibroin hydrogels showed rapid swelling to a 17.9-fold maximum in neutral conditions (pH 7.4) over 12 h (Fig. 1g). The release of FGF1 from the heparinized fibroin hydrogels soaking in the FGF1 solution (Fig. 1f) contained two stages: rapid release where approximately 43.7% of the total FGF1 was released in the first 12 h, then slow release where a cumulative 70% of the total FGF1 in the hydrogel was released over the remaining days. The fibroin hydrogels were rapidly degraded, with 15.5 ± 1.89% of the material lost within the first 2 days (in PBS, pH 7.4), then the degradation rate decreased and a total amount of 32.1 ± 2.36% loss was found within 16 days (Fig. 1h).

### Expressions of cytokine and growth factor in full-thickness skin defects

**Fig. 2:**
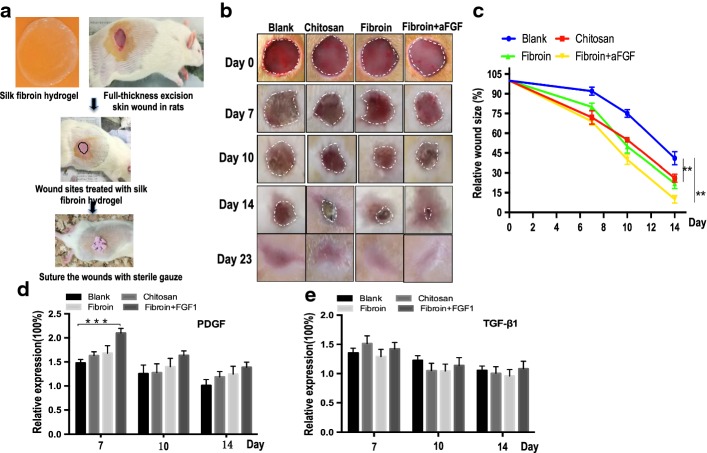
**a** Schematic diagram of silk fibroin hydrogel application for wound healing. The wound was formed by punching a hole through the whole skin of rats. **b** Extent of wound healing on days 7,10 and 14. **c** Measure changes in the percentages of wound closure. **d**, **e** The expression levels of PDGF and TGF-β1 in full-thickness skin defects wound using ELISA at three time points. The relative fold change was ratios between different concentration of growth factors in four operation groups and normal skins cut from rats at different time points. Asterisks indicate statistical significance based on Student’s *t* tests (***P < 0.001)

Growth factors played an important role in regulating the cellular migration and proliferation in the inflammatory phase of wound-healing stage. PDGF and TGF-β were examined by using ELISA at three time points. The relative fold change means different concentrations of growth factors in four operation groups divided by that of normal skins cut from rats. The expression levels of PDGF in silk fibroin hydrogel treated with FGF1 were the highest on day 7, which were significantly higher than those of the chitosan, fibroin hydrogel and blank groups (Fig. 2e) but decreased rapidly on day 10 and 14, with no statistical significant difference among them. Moreover, there was no statistical significant difference for expression of TGF-β1 from day 7 to 14 (Fig. 2f).

### Rat full-thickness skin defects model created and wound size measured

The silk fibroin hydrogels prepared were applied on the full-thickness excisional wounds in rats (Fig. 2a). To visualize changes in wound size over time, photographs of the wound region were taken on days 7, 10, 14 and 23 (Fig. 2b). The wound size reduction rate was calculated by comparing the wound size at each time point with the original wound size on day 0. Initially, the wound size of chitosan- and silk fibroin-treated groups with or without FGF1 was significantly smaller than that of the blank control on day 7, but no significant difference between them was detected. Wound healing in chitosan- and fibroin hydrogel-treated groups were similar on the 10th day after operation. On day 14 postoperation, the wound size reduction rate of the heparinized silk fibroin with FGF1 reached 90.55%, which was significantly higher than that of the untreated blank group (41%, *P* < 0.001) (Fig. 2c). After 23 days, all wounds of full-thickness skin excision were healed completely. In addition, the wounds treated with heparinized hydrogels with FGF1 had significantly reduced scar formation at the visual levels compared with the other groups (Fig. 2b). Our findings showed that the area of the wounds covered with silk biomaterials appeared smooth when compared with chitin wound dressing or blank control on day 23 (Fig. 2b). Taken together, silk fibroin hydrogels with FGF1 have excellent effect in promoting wound healing in rats.

### Histological observation of the wound-healing process

**Fig. 3:**
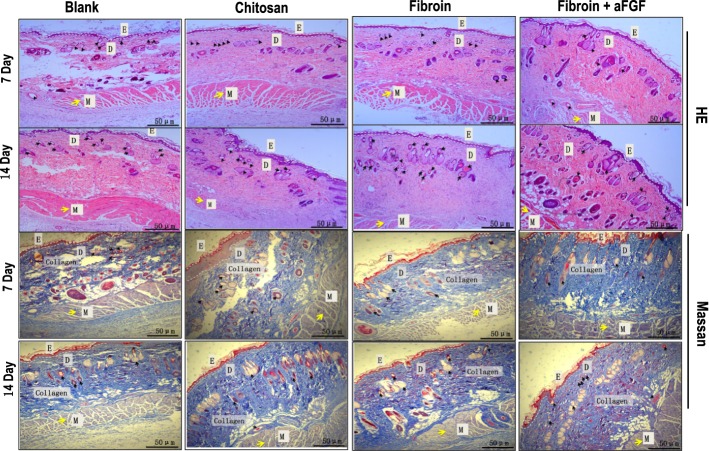
HE staining photographs (upper two panels) and MT staining images (lower two panels) of wounds. Black arrows point to the sites of epidermal differentiation into hair follicles and sebaceous glands and yellow arrows point to the sites of muscle tissue. Blue color represents collagen staining. *D* dermis, *E* epidermis, *M* muscle

To compare the wound-healing effect of silk fibroin hydrogels with chitin wound dressings, we further histologically evaluated the repaired tissue with HE staining at day 7 and 14. On day 7 postoperation, immature granulation tissue under epithelium was evident with loose collagen matrix present in the defect region of the blank control group (Fig. 3). In addition, the structure of the dermis in the blank group was not complete, with large blank areas on day 7 after operation. Compared with the empty group, silk fibroin hydrogel and chitosan dressings can better provide structural support for cell attachment, growth and migration on the wound sites; moreover, the structure of the new full-thickness skin growth healed by fibroin hydrogel and chitosan was intact, containing epidermis (E) and dermis (D) associated with hair follicles, adipose cells and muscle (yellow and black arrows in Fig. 3). After 14 days, well-defined dermal and epidermal layers with abundant collagen deposition and numerous capillaries and sebaceous glands were visible obviously in the wounds treated with fibroin hydrogel and chitosan, whereas in blank control wound, collagen fibers were less densely packed and contained a more porous structure (Fig. 3). Taken together, these data demonstrated that silk fibroin hydrogels with FGF1 could significantly improve wound-healing effect in rats.

### Effects of regenerated silk fibroin hydrogels on L929 cell growth and migration in the presence and absence of FGF1 in culture medium

**Fig. 4:**
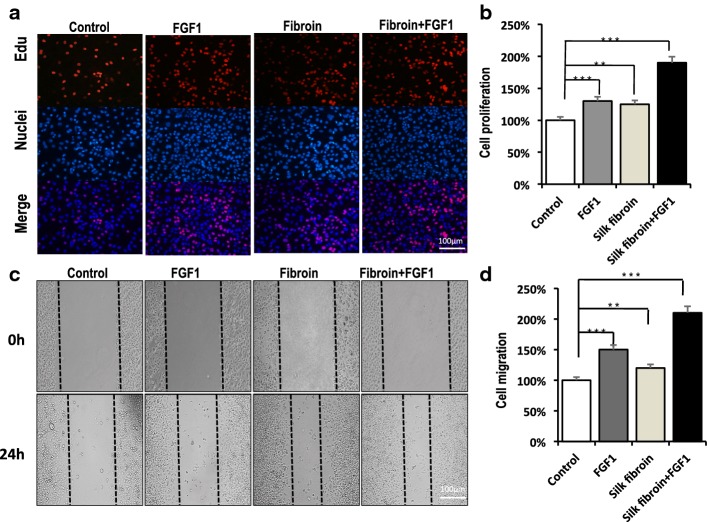
Cell proliferation and wound healing scratch assay. **a**, **b** Proliferation of L929 cells on the surface of the fibroin hydrogels by EdU incorporation. The increased red fluorescence intensity indicated increasing EdU incorporation on the pure fibroin hydrogel or FGF1 solution, compared to blank control. **c**, **d** In vitro wound healing assay. The scratched L929 cells seeded on the surface of pure fibroin hydrogels or TCP dishes were cultured with DMEM medium in the presence or absence of FGF1 for 24 h. The cell numbers in the scratched areas increased differently from four groups after 24 h. The results are representative of three independent experiments. Asterisks indicate statistical significance based on Student’s *t* tests (**P < 0.01, ***P < 0.001)

To understand the mechanism of the wound-healing effects of silk fibroin, firstly the EdU incorporation analysis was performed to visualize the proliferation in L929 cells. The results showed that only a small number of L929 cells from the blank group emitted a weak red fluorescence signal. There was similar luminous intensity between cells in medium with FGF1 or fibroin hydrogel group. However, more cells cultured in silk fibroin hydrogel in the presence or absence of FGF1 in culture medium exhibited intensive red fluorescence signal than the control group (Fig. 4a, b). These results suggested that silk fibroin hydrogel in the presence or absence of FGF1 promoted L929 cell proliferation. Furthermore, wound-healing assay showed more newly generated cells in fibroin hydrogel treated by FGF1 migrated into the scratch regions than the other treated groups (Fig. 4c, d).

## Discussion

Silk fibroin, extensively explored as a promising biomaterial in different forms such as hydrogels, sponges, films and electrospun mats, had excellent biocompatibility, biodegradability and antimicrobial properties to promote wound healing and less scar formation [9, 23–26]. Moreover, many studies showed that silk fibroin produces less inflammation and low immunogenicity [27]. It has been a long time to apply silk fibroin for wound treatment [28]. In the present study, water absorption, swelling ratio and biodegradability of fibroin hydrogels were evaluated at different time points. It was reported that FGF1 release from fibroin hydrogel increased with decrease in fibroin concentration [29, 30]. It is indicated that silk fibroin hydrogel produced a gradual increase and sustained the concentration of FGF1 over a long period, which was suitable for the application of drug delivery. Morphological analysis of the internal structure of fibroin hydrogels showed lamellar, porous and interconnected microstructure, which could provide convenient spaces for cell growth and transport of bioactive molecules. According to the previous research, we selected a lower fibroin concentration to form hydrogel in this study. Prior to the clinical trial, the safety and effectiveness of the fibroin hydrogel should be firstly evaluated in animal model. Considering that human FGF1 has potential therapeutic applications for accelerating wound repair and clinical treatment of chronic non-healing dermal ulcers in diabetes [31–34]. In the present study, full-thickness cutaneous defect on the back of rats were firstly established. Fibroin hydrogels prepared with FGF1 were carried out to cover wound sites to release FGF1 slowly. In this study, heparinized hydrogels loading FGF1 is superior to improve overall wound healing and substantially decrease the time required to achieve total closure, as demonstrated by fast wound closure, re-epithelization and earlier formation of granulation tissue, which were remarkably faster than in the other groups. To better understand wound-healing mechanisms induced by fibroin hydrogel treatment, wound-healing scratch and cell proliferation assay were performed and showed that fibroin hydrogels or FGF1 can facilitate the migration of fibroblast L929 cells, which may provide evidence for cell proliferation and growth in wound sites.

The aim of this study was to investigate the possibility of using wound dressings made of silk fibroin hydrogels functionalized with FGF1 to help the closure of wounds by epidermidalization, dermis, angiogenesis and dense collagen deposition. What is more, silk fibroin has an easy availability from sericulture industry with low cost than the commercially available chitin dressings. All in all, if the aforementioned properties of silk fibroin hydrogel can be utilized and harnessed effectively in the future, there is no doubt that silk fibroin can be developed into new biomaterial and widely used in clinical treatment.

## Conclusion

The aim of this study was to investigate the possibility of using silk fibroin as wound dressing functionalized with FGF1 to help the closure of wounds by increasing wound-healing rate, reepithelialization, dermis formation, collagen synthesis and epidermal differentiation into hair follicles and sebaceous glands compared to chitosan commercially available in market during the whole wound-healing period. The gross study on animal models indicates a visual pattern in which wounds treated with fibroin hydrogels show faster healing. Wound-healing scratch and cell proliferation assay showed that fibroin hydrogels with FGF1 facilitate the migration of fibroblast L929 cells, which is consistent with that obtained in the animal experiment.

Altogether, the results indicated that the functionalized silk fibroin can be developed into new biomaterial and widely used in clinical treatment.

## Methods

### Preparation of silk fibroin hydrogels

Fabrication of the silk fibroin hydrogel was carried out as in the previous published protocols [20]. Cocoons (supplied by Southwest University) were degummed in 0.05% (W/V) Na_2_CO_3_ solution at 85 °C for 30 min. The fibroin extract was then rinsed three times in Millipore water to extract the glue-like sericin proteins and wax. Subsequently, degummed silk fibroin was solubilized in 9.3 M LiBr (lithium bromide) solution for 4 h at 60 °C, yielding a 20% solution (w/v). The mixing silk and LiBr solution obtained is a viscous and honey-like solution. This solution was then filtered through the dialysis membrane (MWCO 3500, Pierce) for 3 days to remove the salts and chemicals and then obtain about 8% (W/V) silk fibroin in aqueous solution, determined by weighing the remaining solid after drying. The silk fibroin solution with an initial concentration of 8 wt% was dialyzed against a 10–25 wt% PEG (10,000 g/mol) solution at room temperature. After 9 h, 21 wt% silk fibroin solution was slowly collected by syringe to avoid excessive shearing and the concentration was determined. 2.3 wt% was prepared by diluting the 8 wt% solutions with distilled water. 5 ml of fibroin solution was prepared in each conical tube and the sonication time was 30 s at the 20% amplitude setting. Then, on immediate transfer of the sonicated solution to a Petri dish and incubation of the plate at 37 °C for 3 days, circular-shaped gels sized 50 mm in diameter and 10 mm in thickness were formed. Hydrogel formation was observed when the sample presented an opaque white color and did not fall when the dish was inverted for 30 s. The silk fibroin hydrogels were then subjected to freeze at − 20 °C for 24 h, and kept at 8 °C for 24 h.

### Heparin immobilization and recombinant human FGF1 treatment of silk fibroin hydrogels

The approach of heparin immobilization was based on previous studies [21, 22]. Heparin sodium salt (Sigma, H3149) was dissolved in a 10% sodium citrate aqueous buffer solution (pH = 4.7) containing EDCI and refrigerated at 4 °C for 2 h to activate the heparin. The fibroin hydrogels prepared were then immersed in the heparin aqueous solution for 24 h at 4 °C to obtain heparin-immobilized hydrogel. After heparin was covalently immobilized on the surface of the hydrogels and washed with citric acid, 0.1% Triton X-100 aqueous solutions, and subsequently rinsed with distilled water in an ultrasonic cleaner for 10 min, it was finally dried in a vacuum dryer and kept in a refrigerator for use. 10 μg of recombinant human FGF1 (from Pepro Tech 100-17A) was centrifuged for 1 min at 1000 rpm prior to opening and dissolved in 5 mM sodium phosphate (pH 8.0) buffer. All the heparinized and non-heparinized fibroin hydrogels were sterilized by a quick spray of 70% ethanol and a exposure under UV light for 15 min under sterile atmosphere. Lastly, heparinized hydrogels were soaked in recombinant human FGF1 solution (1 μg/ml) and allowed to swell for 2 h before using on the wound sites.

### Scanning electron microscopy (SEM)

The fractured sections of silk fibroin hydrogel were cut using a razor blade after freezing in liquid nitrogen (− 196 °C). Fractured and surface sections of samples were vacuum coated with gold/palladium particles using the Polar on SC502 Sputter Coater. The morphology of fibroin hydrogel was examined with a field emission scanning electron microscope (FESEM) Zeiss EVO10 (Carl Zeiss AG, Germany) at 3 kV.

### Water uptake, swelling behavior and porosity measurement for fibroin hydrogels

The water absorption rate of fibroin hydrogels was measured in different time points at room temperature in PBS (pH 7.4). The aqueous solution of the surface of each specimen was promptly removed. The weight change of the specimens was intermittently measured during each time point. The dynamic swelling ratios of the fibroin hydrogels were assayed according to the following equation: Swelling (%) = (Ws − Wd)/Wd × 100. The dried and weighed fibroin hydrogels (Wd) were immersed in PBS, pH 7.4. At different time points, the swollen hydrogels were removed to determine the weight as Ws. The pore size and porosity of hydrogels were measured using the surface area and pore characterization system (ASAP 3020; Micromeritics Instrument CorporationTristar).

### In vitro analysis of FGF1 released from the fibroin hydrogels

The silk fibroin hydrogels soaked in FGF1 solution were added into the six-well plates. Then, 1 ml of PBS (pH 7.4) was added to each well and kept at 37 °C. The supernatant from the wells was transferred to a tube and fresh PBS (pH 7.4) of the same volume was added to the same well at different time points. FGF1 was collecting through the supernatant and then replaced by fresh solution. The amount of released FGF1 was analyzed by enzyme-linked immunosorbent assay kit (ELISA, Westang system, Shanghai, China). The FGF1 release system was carried out at 37 °C.

### Degradation properties of the fibroin hydrogel in vitro

The hydrogel prepared was weighed (W1) in vitro before simulating the degradation testing. The samples were placed in 10 ml PBS solution at 37 °C and weighed at different time points. W2 was the dry weight of composite hydrogel after drying for 12 h. The fibroin hydrogel degradation rate was calculated by the equation of dry weight remaining ratio: DWR = W2/W1 × 100%.

### Cell proliferation assay and in vitro wound-healing assay

L929 cells were kindly provided by Stem Cell Bank, Chinese Academy of Sciences and cultured in DMEM medium with 10% fetal bovine serum (FBS) and 1% penicillin/streptomycin (P/S). The cell proliferation was measured using a Click-iTEdU Imaging Kit (Invitrogen) following the manufacturers’ protocols. The labeled cells with EdU (5-ethynyl-20-deoxyuridine) incorporation were fixed with 3.7% formaldehyde in PBS, and the EdU imaging was detected by fluorescence microscopy at the excitation/emission of 555/565 nm maxima. Analysis was conducted on fluorescence microscopes using single interference filters sets for blue (40,6-diamidino-2-phenylindole (DAPI)). For each interference filter, monochromatic images were acquired and merged using Cyto Vision (Leica Microsystems, Inc.). All cell images captured have the same exposure intensity and magnification. For determination of the percentages of EdU-positive cells, the red-fluorescent marked proliferated cells and (blue-fluorescent) DAPI marked the total cells,and the ratio between them represented the cell proliferation ability. All the slides were treated with the same threshold and the cell number was analyzed with the software Photoshop. Three independent experiments were performed for accuracy and the differences among the groups were compared. The ability of cell migration was determined using the wound-healing scratch assay. Cells were serum starved overnight at 37 °C in a 95% oxygenated tissue culture hood and scratched with a sterile clear tip. An amount of 200 ng FGF1 in 1 ml DMEM medium was used to culture the cells in silk fibroin hydrogels. Initial wounding and the migration of the cells in the scratched area after 24 h were photographically monitored using a microscope (Nikon, Tokyo, Japan) and calculated using Image J software. Measurements were performed in triplicate and repeated independently three times.

### Implants in rats

All SD rats were purchased from Hunan SLAC Laboratory Animal co., LTD (SCXK (Su) 2016-0002 and familiarized with their new environment for a week. All rats (250–350 g) used were male and fed under SPF (specific pathogen-free animal) facility with a 12:12 h light–dark cycle. All surgical procedures were conducted under animal care protocols approved by the Ethics Committee of Chongqing Medical University. Four rats were randomly assigned to each experimental group and distributed by four experimental groups with three time points. Sixty-four rats were randomly divided into four different groups [fibroin (*n* = 4), chitosan (*n* = 4), fibroin + afgf (*n* = 4) and blank group (*n* = 4)] at each time point: 7, 10, 14 and 23 days. After anaesthetization (1.5–3 vol.% Isoflurane), the dorsal surface hairs were shaved and then punched with a sterile 15 mm punch biopsy punch. Blank wound sites were covered with air-permeable Tegarderm™ (3 M, St. Paul, MN) tape, while the other sites were covered with commercial chitin wound dressings or the silk fibroin hydrogels with or without FGF1. The commercial chitosan membranes were chose as the positive control (medical grade, Shanghai HaiChun Biotechnology Corporation). They were used to accelerate the wound healing and induce cell migration and proliferation in the market for many years due to many excellent properties such as low toxicity, good biocompatibility, antibacterial activity and so on. All rats were intraperitoneally injected with penicillin (20,000 U/kg/days) daily for 3 days. Rats were killed by CO_2_ exposure after each time point and samples were collected for histological examination. The images of the skin wounds in this study were captured using digital camera and imported onto Image J (v1.8.0) software. The extent of wound healing is expressed as the percentage of area remaining exposed. Wound size (%) = [*R*(7,10,14)/*R*(0)] × 100, where *R*(0) and *R*(7,10,14) represents the exposed wound area at postoperative days 0 and 7, 10, and 14, respectively.

### Histological evaluation of the wounded skins healing in rats

The silk fibroin hydrogels were cut into round shape and glued to the wound sites of rats. Rats were killed for histological analysis after healing for 7, 10 and 14 days, respectively. Specimens were harvested at these three time points and immediately fixed in 10% formalin solution at room temperature for at least 24 h. Subsequently, paraffin-embedded tissue sections of 10 μm thickness were obtained by using HE and MT staining for demonstration of skin architecture and collagen fibers by standard procedures.

### The expression analysis of PDGF and TGFβ released from the skin tissues of wound healing

Rats were killed for assessment of PDGF and TGFβ at the indicated time points after healing by chitosan wound dressing and fibroin hydrogels with or without FGF1 for 7, 10 and 14 days. New skin tissues were disrupted using a Mini-BeadBeater-16 (Biospec) and homogenized with a tissue homogenizer following centrifugation (750*g*, 4 °C) for 10 min after lysing in radio-immunoprecipitation (RIPA) buffer from Santa Cruz Biotechnology (Santa Cruz, CA, USA). Total protein concentration was determined by BCA Protein Assay Kit (Thermo Scientific). The samples were stored at − 80 °C and used for ELISA determination.

### Statistical analysis

GraphPad Prism v. 5.0 software (GraphPad Inc.; San Diego, CA, USA) was used for all the statistical analyses. Data represent mean ± SD. The statistical significance was assessed using two tailed Student’s *t* tests (*P < 0.05, **P < 0.01, ***P < 0.001).

## Data Availability

Data sharing not applicable to this article as no datasets were generated or analyzed during the current study.

## Abbreviations

FGF1: fibroblast growth factor-1
PDGF: platelet-derived growth factor
TGF-β1: transforming growth factor-β1
ELISA: enzyme-linked immunosorbent assay
HE: hematoxylin and eosin
EDCI: 1-ethy-3-(-3dimethylamino-propyl) carbodiimide
MT: Masson’s trichrome
SD: Sprague–Dawley
FBS: fetal bovine serum
SEM: scanning electron microscopy
EdU: 5-ethynyl-20-deoxyuridine
LiBr: lithium bromide
SPF: specific pathogen-free animal
